# Quality of care for children with severe disease in the Democratic Republic of the Congo

**DOI:** 10.1186/s12889-019-7853-3

**Published:** 2019-12-02

**Authors:** Emma Clarke-Deelder, Gil Shapira, Hadia Samaha, György Bèla Fritsche, Günther Fink

**Affiliations:** 1 Harvard T. H. Chan School of Public Health, Boston, USA; 2 The World Bank, Washington, DC USA; 3 Swiss Tropical and Public Health Institute and University of Basel, Socinstrasse 57, 4051 Basel, Switzerland

**Keywords:** child health, quality of care, infectious disease

## Abstract

**Background:**

Despite the almost universal adoption of Integrated Management of Childhood Illness (IMCI) guidelines for the diagnosis and treatment of sick children under the age of five in low- and middle-income countries, child mortality remains high in many settings. One possible explanation of the continued high mortality burden is lack of compliance with diagnostic and treatment protocols. We test this hypothesis in a sample of children with severe illness in the Democratic Republic of the Congo (DRC).

**Methods:**

One thousand one hundred eighty under-five clinical visits were observed across a regionally representative sample of 321 facilities in the DRC. Based on a detailed list of disease symptoms observed, patients with severe febrile disease (including malaria), severe pneumonia, and severe dehydration were identified. For all three disease categories, treatments were then compared to recommended case management following IMCI guidelines.

**Results:**

Out of 1180 under-five consultations observed, 332 patients (28%) had signs of severe febrile disease, 189 patients (16%) had signs of severe pneumonia, and 19 patients (2%) had signs of severe dehydration. Overall, providers gave the IMCI-recommended treatment in 42% of cases of these three severe diseases. Less than 15% of children with severe disease were recommended to receive in-patient care either in the facility they visited or in a higher-level facility.

**Conclusions:**

These results suggest that adherence to IMCI protocols for severe disease remains remarkably low in the DRC. There is a critical need to identify and implement effective approaches for improving the quality of care for severely ill children in settings with high child mortality.

## Background

Global child mortality has decreased by more than 50% since 1990, from 85 deaths per 1000 live births in 1990 to 38 deaths per 1000 live births in 2016 [1]. However, child mortality in sub-Saharan Africa remains high, at an estimated 77 deaths per 1000 live births [1].

In the mid-1990s, the World Health Organization (WHO) introduced the Integrated Management of Childhood Illnesses (IMCI) strategy to improve quality of care for sick children under the age of five.

Over two decades have passed since the introduction of IMCI, and a growing literature documents persistently low quality care for sick children in low- and middle-income countries [2–6]. For example, in a recent study in rural India, only 13% of providers recommended the correct treatment for patients with pneumonia and none recommended the correct treatment for patients with diarrhea [3].

While lack of adherence to clinical guidelines is likely a lesser concern for mild disease, lack of a timely and appropriate clinical response may be fatal in the case of severe disease. To date, evidence on the management of severe disease remains scarce. In one of the few studies in this area, virtually none of the patients classified as severe cases by health workers in Tanzania got the recommended treatment package [7].

In this study, we measured quality of care for children with severe disease in the Democratic Republic of the Congo (DRC). The DRC is the second largest country in sub-Saharan Africa with an estimated population of 81 million and an under-five mortality rate of 91 deaths per 1000 live births [8].

To assess the quality of care for children with severe disease, we developed a new direct observation approach for under-5 care. Rather than re-assessing the child upon exit [4, 9], we used tablets to collect information on both the diagnostic procedures performed by providers and the disease and symptom information collected by the provider through these procedures. These symptoms and diagnostic test results were then used to classify cases.

## Methods

### Study design

We conducted a cross-sectional observational study relying on detailed direct observation measures collected by trained study staff during facility visits by children under five and their caregivers. Our objectives were threefold: 1) to evaluate average provider performance in assessing patients for signs of severe disease, 2) to use available symptom data to classify under-5 visits into cases of severe disease following IMCI guidelines, and 3) to evaluate average quality of treatment for severe cases.

### Setting

This study was conducted in five provinces of the DRC: Equateur, Bandundu, Katanga, North Kivu, and South Kivu.^1^ The leading causes of under-five mortality in the DRC are neonatal disorders (33%), lower respiratory infection (15%), congenital birth defects (9%), diarrhea (10%), and malaria (7%) [10]. The DRC has experienced recurrent conflicts since the 1990s and ranks in the bottom ten countries worldwide on the Human Development Index, a composite measure of health, education, and standard of living [11]. The DRC also ranks in the bottom five countries worldwide in terms of healthcare expenditure per capita: in 2016, the estimated expenditure per capita was $20.52 [8].

Since 2004, the DRC’s national protocol for the management of severe childhood illness has been based on the WHO’s IMCI strategy. However, implementation of the IMCI strategy in the DRC has been fragmented and delayed: a recent WHO report cites numerous challenges in implementation, including insufficient numbers of IMCI trainers, and the fragmentation of health sector funding in different regions of the country [12].

The DRC has also scaled up the WHO’s strategy for community care, known as integrated Community Case Management (iCCM). However, a household survey conducted in the catchment areas for our sampled facilities shows that 41% of children with any sickness in the two weeks preceding the survey were taken to a health facility while only 1% of sick children were brought to community health workers [8].

The DRC has unique challenges in the management of its health workforce. There is a highly unequal distribution of health providers across the country, with a plethora of providers in areas with high population density and a lack of certain cadres of providers in more remote areas. Many health providers in the DRC are trained in programs that are not regulated by the Ministry of Health [13].

### Sampling and Participants

We analyzed baseline data collected between June 2015 and March 2016 for the evaluation of World Bank Results-Based Financing (RBF) programs in the DRC. Because these data were collected before the RBF programs were implemented, the programs are relevant only to the sampling of facilities, not to the provider behavior that we observed.

For the baseline survey conducted for this project, 266 health centers and 80 hospitals were randomly selected for participation in the study. Two health centers were randomly selected from each of the 133 health zones in the study provinces, from a list of 6474 health centers provided by the Ministry of Health. The hospitals in the sample were selected from the 82 health zones in the study sample that had hospitals. The sampled health centers and hospitals reported an average of 101 patients under-5 in the preceding month (SD = 125). The reported volume was higher in health centers (103 patients under-5 per month) than hospitals (86 patients under-5 per month).

Children under two months of age were omitted from this analysis because the sample size for this age group was small and the treatment protocol is different from the protocol for older children. Some of the sampled health centers did not have any curative visits with patients under five on the day of data collection. The resulting sample after omitting 25 facilities without eligible patients included 321 facilities.

### Procedures

Selected facilities were visited by the study team to introduce the study (which was directly supported by the Ministry of Health). Trained enumerators then followed between one and five under-5 patients visits at each facility, selected based on availability when the enumerator was ready to conduct this part of the data collection process, conditional on the consent of caregivers.

During the patient’s visit, enumerators recorded all diagnostic tests conducted and all questions asked by the provider as done in standard direct observation tools. In addition, enumerators recorded all information collected by the provider through these diagnostic tests, such as duration of symptoms, danger signs, body temperature, and any other test result. In case enumerators did not manage to understand or note test results during the visit, this information was obtained from the provider after the patient left. An exit interview was conducted with the patient’s caregiver, during which the enumerator asked the caregiver to show them the prescriptions and medications provided during the consultation or purchased afterwards.

The data were collected using structured checklists and multiple-choice questionnaires, which were developed specifically for this project, and programmed onto tablets using Open Data Kit (ODK) software. The checklist items were recorded quantitatively. For example, the checklist included an item for whether the provider asked about the duration of the child’s fever. If the observer selected “yes,” they were prompted to record the caregiver’s response about the duration in days or hours. Some checklist items (e.g. the medications provided to the patient) were completed using free text response. The data collection tools used in this study are available online [14].

### Data sources/measurement

We used data from direct observations of 1249 provider consultations with sick children under the age of five. We merged this dataset with exit interview data using unique identification codes assigned to patients and facilities, as well as information on patient characteristics. 6% of cases were not successfully merged either because caregivers were not available for exit interviews (4%) or because of data entry error resulting in duplicated or missing identification codes (2%). We focus our analysis on 1180 cases that were successfully merged.

Using the merged dataset, we followed IMCI guidelines to classify cases of severe febrile disease (including malaria), severe pneumonia and other respiratory infections, and severe dehydration as the most common causes of child mortality (among those who survive infancy) in the DRC [10].

Table 1 summarizes the IMCI guidelines for classification and treatment of these three severe diseases, as described in the 2014 IMCI chart booklet [15]. Our coding followed these guidelines, with one exception: because our dataset did not include information on the symptom of neck stiffness, we could not include this in the diagnosis of very severe febrile disease. In our analysis, very severe febrile disease was defined as the presence of fever and any “general danger sign.” which likely under-estimates the actual prevalence of very severe febrile disease.

**Table 1:** Severe disease classifications and recommended treatments from the 2014 IMCI codebook

Severe disease classifications	Signs	Recommended treatments
Severe pneumonia or other respiratory infection	(1) Cough or difficulty breathing AND (2) Any *general danger sign* (lethargy or loss of consciousness, convulsions, inability to drink or breastfeed, vomiting all food) OR stridor in calm child	• Give first dose of an appropriate antibiotic. • Refer urgently to hospital. If referral is not possible, manage the child as described in the pneumonia section of the national guidelines or as in the WHO Pocket Book for hospital care for children.
Severe dehydration	(1) Diarrhea AND (2) Two or more of the following: lethargic or unconscious, sunken eyes, not able to drink or drinking poorly, skin pinch goes back very slowly	• If child has no other severe classification, give fluid for severe dehydration. • If child has another severe classification, refer urgently to hospital with mother giving frequent sips of ORS on the way. • Advise the mother to continue breastfeeding. • If child is 2 years or older and there is cholera in your area, give antibiotic for cholera.
Very severe febrile disease (including malaria)	(1) Fever AND (2) Any *general danger sign* OR stiff neck	• Give first dose of artesunate or quinine for severe malaria. • Give first dose of an appropriate antibiotic. • Treat the child to prevent low blood sugar. • Give one dose of paracetamol in clinic for high fever (38.5 C or above). • Refer urgently to hospital.

If, conditional on a child’s main symptom(s) (cough, fever, or diarrhea), the diagnostic testing conducted was insufficient to confirm or rule out severe disease, we coded this as a “potential” severe disease case, but did not include it in our main analysis.

We divided severe febrile disease cases into three categories: confirmed malaria (patients with a positive malaria test), untested for malaria, and other fevers (patients with a negative malaria test).

### Potential Bias

Given that providers knew they were observed during the consultations, there is a potential for Hawthorne effects, whereby healthcare providers change their behavior in the presence of an observer. We would expect Hawthorne effects to bias our findings towards higher quality care. In addition, we were only able to classify severe disease cases if providers conducted sufficient examinations of patients. Missing data due to insufficient testing by providers may lead us to overestimate the rate of correct diagnosis if providers with less information on average make worse diagnostic decisions. Therefore, average quality of care reported in this paper is likely an upper bound on the true quality.

### Statistical methods

We started our analysis with an evaluation of providers’ assessments of patients. Using data from direct observations, we investigated whether providers conducted sufficient diagnostic testing to confirm or rule out cases of severe disease, conditional on patients’ main symptoms.

We then described the severe disease cases and investigated provider performance in managing these cases. We first analyzed whether providers diagnosed the type of disease (malaria, pneumonia or other respiratory infection, or dehydration) and the severity correctly. Our analysis of the diagnosis of disease severity focused only on respiratory infection and dehydration because we did not have data on providers’ diagnosis of febrile disease severity. Next, we analyzed treatment. Specifically, we looked at provider decisions to refer patients to another facility or to in-patient care, and at medications provided. While the IMCI guidelines recommend referral to a hospital for all patients with severe diseases, we look at both referral and recommendation for in-patient care because referral may not be feasible in all severe cases. Given the many rivers and lakes in the study area, the route from a health center to a higher-level facility might require several modes of transportation, and the best intensive care option for patients may be local in-patient treatment.

We used an inclusive coding approach for medications, considering all medications that were given to the patient in the facility, prescribed to the patient, or purchased by the caregiver during the visit. Our coding followed the IMCI protocol, with two exceptions due to data limitations: for treatment of severe dehydration, we did not include the recommendation that the mother continue breastfeeding, and, for treatment of severe febrile disease, we did not include treatment for low blood sugar. The IMCI recommended treatment for severe dehydration depends on whether the patient is in an area with cholera: due to potential cholera risk in the DRC, we coded the recommended treatment as fluids and antibiotics.

Finally, we stratified our analysis by factors that could potentially be associated with higher compliance with the IMCI guidelines based on our review of related studies, including geographic area (urban vs. rural), province, health facility type, provider cadre, provider experience with IMCI training, whether the provider diagnosed the case as severe, and the number of IMCI-determined severe diseases the patient had. We distinguished between three types of facilities: hospitals, referral health centers (which provide a package of services similar to hospitals and typically have a doctor on staff), and regular health centers.

#### Missing data

Diagnostic information was missing from 6 (3%) of the IMCI-determined severe pneumonia cases in the sample, but not from any of the IMCI-determined severe febrile disease or severe dehydration cases. Treatment information was missing from 41 (11%) of the cases in the severe disease sample. In our analysis, we assumed that both diagnostic information and treatment information were missing completely at random.

## Results

**Fig. 1:**
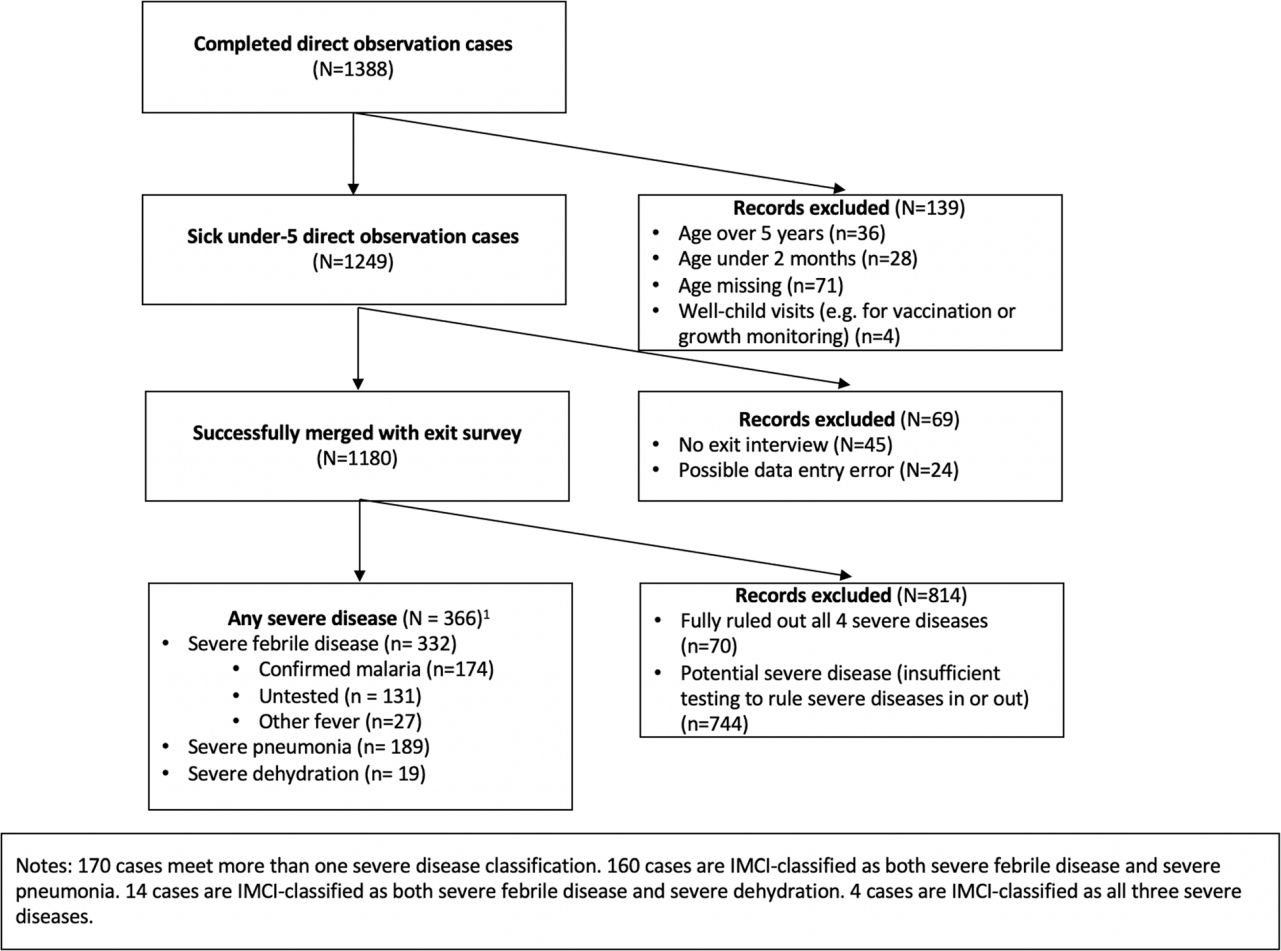
Sample Flow Chart

We identified 366 IMCI-classified severe disease cases among the 1180 observations of sick under-5 cases that were successfully merged (Fig. 1). In 744 cases (63%), providers did not conduct enough tests or assessments to sufficiently determine whether the patient had severe disease. This percentage was similar across the three diseases, ranging from 65% for patients presenting with fever to 69% for patients presenting with diarrhea. The most commonly omitted questions or checks resulting in classification gaps were: checking for stridor among patients with cough, and asking caregivers about loss of consciousness and convulsions among patients with or diarrhea.

Based on observed symptoms, 189 cases (16%) met the IMCI classification for severe pneumonia or other respiratory infection, 19 cases (2%) met the IMCI classification for severe dehydration, and 332 cases ( 28% of the observation sample) met the IMCI classification for severe febrile disease. 160 patients (14%) had signs of both severe pneumonia and severe febrile disease.

The large majority of patients with severe disease (94.5%) were seen at health facilities in rural areas (Table 2). Approximately two thirds (67.5%) visited health centers rather than hospitals: 10.1% visited referral health centers and 57.4% visited regular (non-referral) health centers. The majority of patients (71.9%) were assessed by a nurse. Despite the fact that training on IMCI in DRC began in 2004 [12], only 35.8% of patients were seen by a provider who had ever been trained on IMCI protocols and only 12.5% of patients were seen by a provider who had recently been trained on IMCI protocols.

**Table 2:** Description of sample

	All (%)	Severe febrile disease (including malaria) (%)	Severe febrile disease (non-malaria) (%)	Severe pneumonia (%)	Severe dehydration (%)
N	366	305	27	189	19
	*N (%)*	*N (%)*	*N (%)*	*N (%)*	*N (%)*
Urban vs. rural					
Urban	20 (5.5)	20 (6.6)	0 (0.0)	8 (4.2)	0 (0.0)
Rural	346 (94.5)	285 (93.4)	27 (100.0)	181 (95.8)	19 (100.0)
Province					
Bandundu	88 (24.0)	73 (23.9)	4 (14.8)	38 (20.1)	5 (26.3)
Equateur	101 (27.6)	81 (26.6)	6 (22.2)	55 (29.1)	5 (26.3)
Katanga	72 (19.7)	61 (20.0)	4 (14.8)	36 (19.0)	2 (10.5)
North Kivu	21 (5.7)	17 (5.6)	2 (7.4)	15 (7.9)	2 (10.5)
South Kivu	84 (23.0)	73 (23.9)	11 (40.7)	45 (23.8)	5 (26.3)
Health facility type					
Hospital	119 (32.5)	104 (34.1)	4 (14.8)	56 (29.6)	11 (57.9)
Referral health center	37 (10.1)	30 (9.8)	5 (18.5)	14 (7.4)	2 (10.5)
Regular health center	210 (57.4)	171 (56.1)	18 (66.7)	119 (63.0)	6 (31.6)
Provider type					
Doctor	92 (25.1)	83 (27.2)	3 (11.1)	43 (22.8)	10 (52.6)
Nurse	263 (71.9)	212 (69.5)	23 (85.2)	137 (72.5)	9 (47.4)
Other	11 (3.0)	10 (3.3)	1 (3.7)	9 (4.8)	0 (0.0)
Provider training on IMCI					
Trained	131 (35.8)	107 (35.1)	11 (40.7)	65 (34.4)	5 (26.3)
Not trained	235 (64.2)	198 (64.9)	16 (59.3)	124 (65.6)	14 (73.7)
Patient sex					
Male	200 (54.6)	170 (55.7)	13 (48.1)	98 (51.9)	13 (68.4)
Female	166 (45.4)	135 (44.3)	14 (51.9)	91 (48.1)	6 (31.6)
Patient age					
2–12 months	113 (30.9)	83 (27.2)	11 (40.7)	65 (34.4)	13 (68.4)
1–2 years	101 (27.6)	89 (29.2)	6 (22.2)	60 (31.7)	4 (21.1)
2–3 years	73 (19.9)	65 (21.3)	6 (22.2)	29 (15.3)	0 (0.0)
3–4 years	54 (14.8)	46 (15.1)	3 (11.1)	25 (13.2)	1 (5.3)
4–5 years	25 (6.8)	22 (7.2)	1 (3.7)	10 (5.3)	1 (5.3)

Notes: Column 1 shows descriptive statistics for all patients with IMCI signs of severe febrile disease, severe pneumonia, or severe dehydration. Column 2 shows descriptive statistics for patients with signs of severe febrile disease and a positive or missing malaria test, who are classified as “severe febrile disease (including malaria).” Column 3 shows descriptive statistics for patients with signs of severe febrile disease and a negative malaria test, who are classified as “severe febrile disease (other).” Columns 4 and 5 show descriptive statistics for patients with signs of severe pneumonia and severe dehydration, respectively. Patients with multiple severe disease indications are shown in multiple columns

**Fig. 2:**
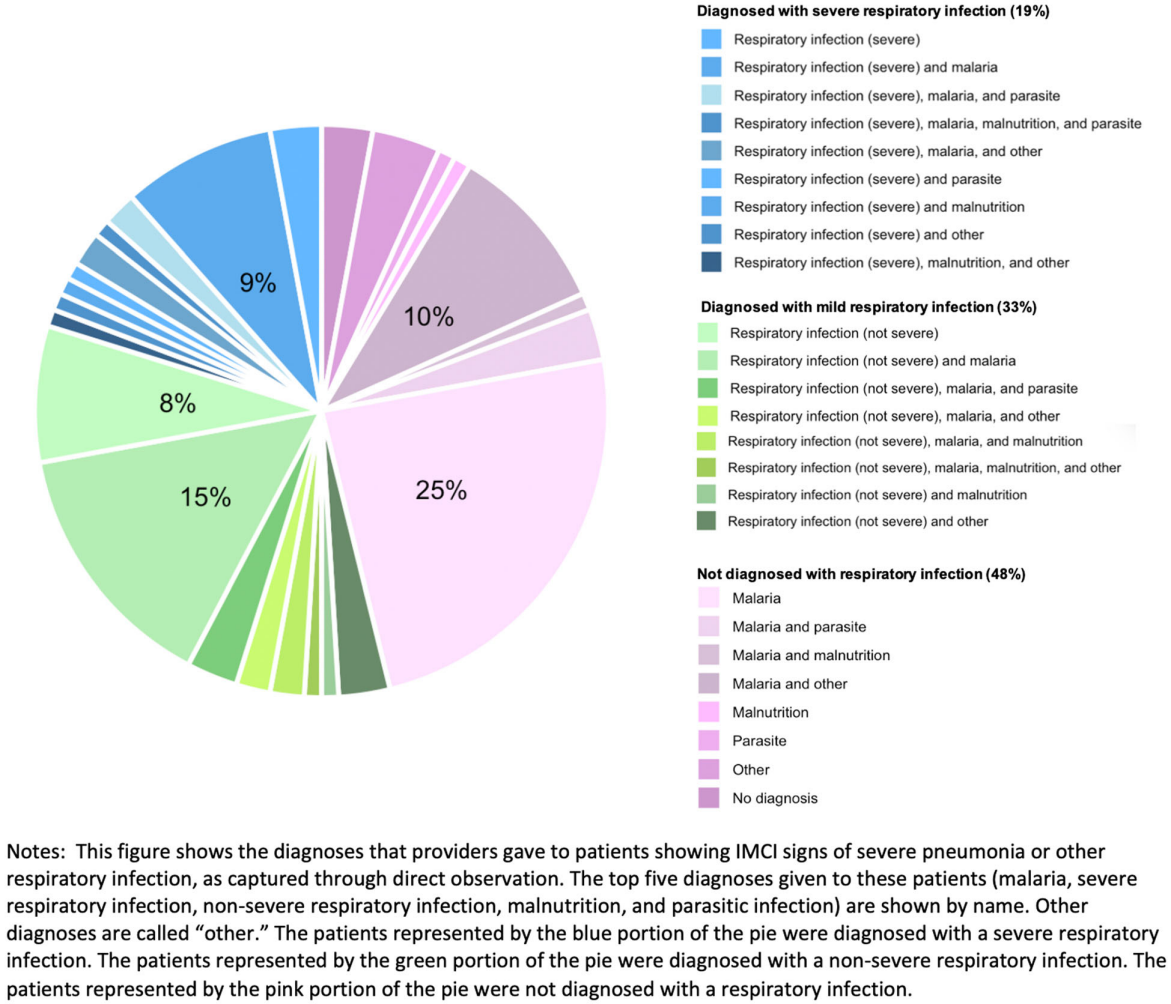
Distribution of Diagnoses for Cases with Severe Pneumonia or Other Severe Respiratory Infection

189 patients (16% of the full sample and 52% of severely ill children) had the IMCI signs of severe pneumonia or other respiratory infection. The most typical signs of severe respiratory infections were the presence of cough or difficulty breathing and: vomiting everything (54% of these cases), inability to drink (29%), or loss of consciousness (23%). Among these patients, 95 (52%) were diagnosed with respiratory infections, and 34 (19%) were diagnosed with severe respiratory infections (Fig. 2). 114 (69%) received antibiotics, the recommended treatment for severe pneumonia or other severe respiratory infection (Table 3). 13% of patients with signs of severe respiratory infection were recommended to some form of in-patient care, either in the facility they visited or through referral to a higher-level facility. Among patients visiting hospitals, 23% were recommended to in-patient care in the facility they visited and 4% were referred elsewhere. Among patients visiting health centers (combining the two health center categories together), 3% were recommended to in-patient care in the facility they visited and 4% were referred elsewhere. Patients diagnosed with severe infections were no more likely to receive the correct treatment or the correct recommendation for in-patient care than other patients.

**Table 3:** Diagnosis and treatment of severe pneumonia or other severe respiratory infection

		Proportion of IMCI-determined severe pneumonia (or other severe respiratory infection) cases in which providers …				
		Diagnosed with pneumonia or other respiratory infection	Diagnosed with severe pneumonia or other severe respiratory infection	Prescribed antibiotics	Recommended inpatient care (in the facility where the visit took place)	Referred to another facility
	*N*	*N (%)*	*N (%)*	*N (%)*	*N (%)*	*N (%)*
All	189	95 (52)	34 (19)	114 (69)	17 (9)	7 (4)
Province						
Bandundu	38	12 (32)	5 (14)	20 (59)	4 (11)	2 (5)
Equateur	55	40 (74)	16 (30)	33 (82)	2 (4)	0 (0)
Katanga	36	13 (41)	4 (12)	22 (61)	2 (6)	2 (6)
North Kivu	15	8 (53)	3 (20)	10 (67)	3 (20)	2 (13)
South Kivu	45	22 (49)	6 (13)	29 (71)	6 (13)	1 (2)
*p-value*		< 0.001	0.134	0.190	0.216	0.142
Urban vs. rural						
Urban	8	2 (25)	1 (12)	4 (57)	0 (0)	0 (0)
Rural	181	93 (53)	33 (19)	110 (69)	17 (9)	7 (4)
*p-value*		*0.146*	*0.681*	*0.504*	*0.366*	*0.573*
Facility type						
Hospital	56	26 (46)	7 (12)	28 (60)	13 (23)	2 (4)
Referral health center	14	6 (43)	1 (7)	10 (77)	2 (14)	0 (0)
Other health center	119	63 (56)	26 (23)	76 (72)	2 (2)	5 (4)
*p-value*		0.617	0.179	0.267	< 0.001	0.735
Training on IMCI protocols						
Trained	65	28 (45)	8 (13)	39 (65)	7 (11)	2 (3)
Not trained	124	67 (55)	26 (21)	75 (71)	10 (8)	5 (4)
*p-value*		0.154	0.142	0.446	0.54	0.743
Provider type						
Doctor	43	18 (42)	6 (14)	23 (68)	10 (23)	0 (0)
Nurse	137	69 (53)	24 (18)	84 (68)	7 (5)	7 (5)
Other	9	8 (89)	4 (44)	7 (78)	0 (0)	0 (0)
*p-value*		0.037	0.093	0.833	0.001	0.255
Diagnosis						
Diagnosed: respiratory infection, not severe	62	–	–	48 (89)	5 (8)	1 (2)
Diagnosed: respiratory infection, severe	28	–	–	20 (83)	2 (6)	1 (3)
Not diagnosed with respiratory infection	99	–	–	46 (52)	10 (11)	5 (5)
*p-value*		*–*	*–*	< 0.001	0.687	0.483
Number of IMCI-determined SDs						
1	29	17 (61)	6 (21)	19 (73)	0 (0)	0 (0)
2	156	76 (50)	28 (19)	92 (67)	17 (11)	7 (4)
3	4	2 (50)	0 (0)	3 (100)	0 (0)	0 (0)
*p-value*		0.623	0.604	0.422	0.14	0.468

Notes: This table shows quality of care for the subset of patients who met the IMCI classification for severe pneumonia or other respiratory infection based on observed symptoms. The columns represent different steps in providing quality of care for severe respiratory infections, and the rows represent different subsets of the data. The results shown are the number of patients and percent of patients in each subgroup for whom each step was taken (based on direct observation data and caregiver exit interviews). *P*-values were calculated using a chi-square test comparing means in the subgroups presented. For diagnosis and treatment, missing data are treated as missing completely at random. There are 6 cases missing information on diagnosis and 23 cases missing observation on treatment; these are omitted from the percentages presented in the table

**Fig. 3:**
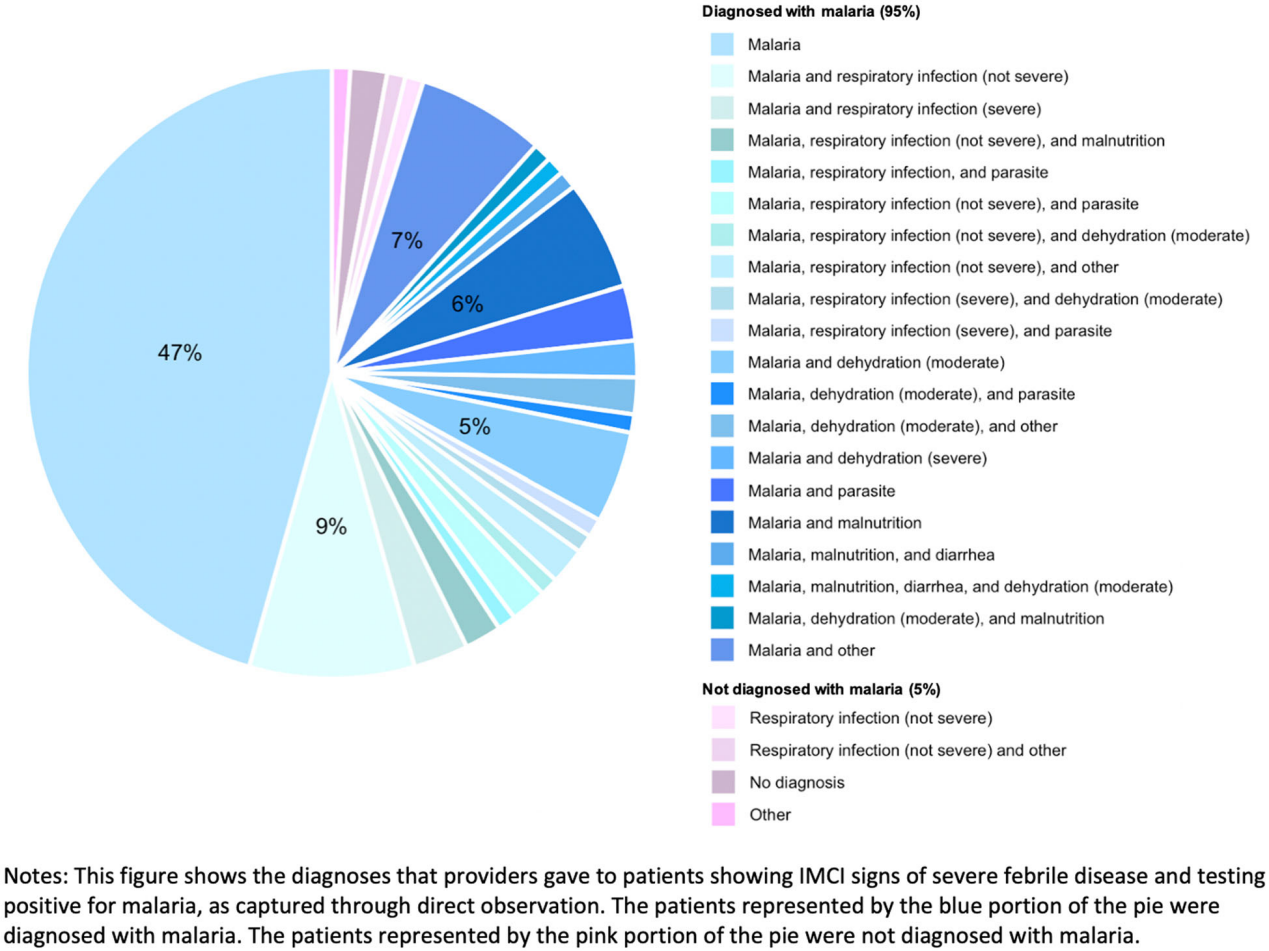
Diagnoses for patients with severe febrile illness

Among patients meeting the IMCI severe febrile disease classification, 54.0% tested positive for malaria, 8.1% tested negative for malaria, and 39.4% were not tested (an 86.6% positive rate among those who were tested). 33% of patients with severe febrile disease (confirmed malaria) were given the correct treatments, including antibiotics, anti-malarial drugs, and, if they had a high fever, paracetamol (Table 4). The majority (95%) of these patients were correctly diagnosed with malaria (Fig. 3). We did not find significant differences in correct treatment rates between providers who had been trained on IMCI protocols and those who had not. 21% of severe febrile disease cases with confirmed malaria were referred to in-patient care, either in the facility they visited or elsewhere. As with the other forms of severe disease, patients visiting hospitals were more likely to be recommended to in-patient care than patients visiting health centers.

**Table 4:** Diagnosis and treatment of severe febrile illness (confirmed malaria)

		Proportion of severe febrile disease with confirmed malaria cases in which providers …						
		Diagnosed with malaria	Prescribed antibiotics	Prescribed anti-malarials	Prescribed paracetamol	Prescribed the correct treatments (based on temperature)	Recommended in-patient care (where the visit took place)	Referred to another facility
	*N*	*N (%)*	*N (%)*	*N (%)*	*N (%)*	*N (%)*	*N (%)*	*N (%)*
All	174	166 (95)	82 (50)	140 (85)	92 (56)	54 (33)	28 (16)	8 (5)
Province								
Bandundu	36	33 (92)	21 (62)	30 (88)	14 (41)	14 (41)	10 (28)	2 (6)
Equateur	29	29 (100)	12 (46)	22 (85)	10 (38)	9 (35)	1 (3)	0 (0)
Katanga	47	46 (98)	24 (51)	37 (79)	19 (40)	12 (26)	5 (11)	2 (4)
North Kivu	11	9 (82)	5 (45)	8 (73)	8 (73)	3 (27)	2 (18)	2 (18)
South Kivu	51	49 (96)	20 (43)	43 (91)	41 (87)	16 (34)	10 (20)	2 (4)
*p-value*		*0.096*	*0.535*	*0.343*	*< 0.001*	*0.684*	*0.071*	*0.189*
Urban vs. rural								
Urban	8	7 (88)	5 (62)	5 (62)	3 (38)	2 (25)	0 (0)	0 (0)
Rural	166	159 (96)	77 (49)	135 (86)	89 (57)	52 (33)	28 (17)	8 (5)
*p-value*		*0.277*	*0.461*	*0.071*	*0.375*	*0.602*	*0.207*	*0.528*
Facility type								
Hospital	65	64 (98)	35 (61)	48 (84)	22 (39)	23 (40)	22 (34)	2 (3)
Referral health center	17	15 (88)	3 (18)	13 (76)	11 (65)	1 (6)	1 (6)	0 (0)
Other health center	92	87 (95)	44 (48)	79 (87)	59 (65)	30 (33)	5 (5)	6 (7)
*p-value*		*0.174*	*0.006*	*0.548*	*< 0.001*	*0.029*	*< 0.001*	*0.384*
Training on IMCI protocols								
Trained	68	66 (97)	34 (51)	57 (85)	32 (48)	22 (33)	11 (16)	4 (6)
Not trained	106	100 (94)	48 (49)	83 (85)	60 (61)	32 (33)	17 (16)	4 (4)
*p-value*		*0.406*	*0.825*	*0.947*	*0.221*	*0.947*	*0.981*	*0.52*
Provider type								
Doctor	52	50 (96)	23 (51)	40 (89)	19 (42)	15 (33)	18 (35)	0 (0)
Nurse	117	111 (95)	57 (50)	96 (83)	69 (60)	37 (32)	9 (8)	8 (7)
Other	5	5 (100)	2 (40)	4 (80)	4 (80)	2 (40)	1 (20)	0 (0)
*p-value*		*0.828*	*0.895*	*0.664*	*0.012*	*0.947*	*< 0.001*	*0.131*
Number of IMCI-determined SDs								
1	89	85 (96)	31 (35)	78 (89)	48 (55)	19 (22)	14 (16)	2 (2)
2+	85	81 (95)	51 (66)	62 (81)	44 (57)	35 (45)	14 (16)	6 (7)
*p-value*		*0.947*	*< 0.001*	*0.149*	*0.776*	*0.002*	*0.131*	*0.895*

Notes: This table shows quality of care for the subset of patients who met the IMCI classification for severe febrile disease based on observed symptoms and also tested positive for malaria. The columns represent different steps in providing quality of care for severe febrile disease, and the rows represent different subsets of the data. The results shown are the number of patients and percent of patients in each subgroup for whom each step was taken (based on direct observation data and caregiver exit interviews). *P*-values were calculated using a chi-square test comparing means in the subgroups presented. There are 9 cases with missing information on the treatments given; we assume that this information is missing completely at random

Results for patients with severe dehydration are shown in Additional file 1: Figure S1 and Table S1. Results for patients with severe febrile disease who were not tested for malaria and who tested negative for malaria are shown in Additional file 1: Tables S2 and S3, respectively.

## Discussion

The results of this study present a stark picture of current clinical diagnosis and treatment of severely ill patients under the age of five in health facilities in the DRC. Our results suggest that critical information required for appropriate diagnosis is not collected by a majority of providers, which makes accurate diagnosis unlikely from the outset. Second, and maybe worse, even those providers who collect the most relevant pieces of information often fail to correctly diagnose health problems and severity. These limitations seem to be particularly pronounced for severe pneumonia or other respiratory infections, where only half of providers correctly classify cases as respiratory infections and less than one fifth of providers correctly classify them as severe. Maybe most troubling, even when the correct diagnosis is made – as is generally only the case for malaria – providers often fail to give appropriate medication. Last, across all three diseases, less than one fifth of severely ill children were recommended to urgently needed inpatient care, either in the facility they visited or at a higher-level facility.

These findings are consistent with a growing literature showing low adherence to IMCI protocols across countries [5, 16, 17]. Our study builds on this literature by bringing a focus to the low quality of care for cases of IMCI-classified severe disease.

The low rates of referral to in-patient care observed in this study may be explained in part by the geographic inaccessibility of the health facilities in the sample. However, this does not explain the low rate of in-patient care in hospitals and referral health centers, which have the capacity to provide this care. In our sample, only 24% of severely ill patients seen at hospitals and 14% of severely ill patients seen at referral health centers received in-patient care. The observed variation between hospitals and health centers may be driven by facility-level capacity to provide in-patient care.

We find some differences across provinces in quality of care. For example, patients with severe pneumonia or severe malaria in Equateur are more likely to be diagnosed correctly than patients with these conditions in other provinces in the study. However, for other indicators, other provinces perform better. The provinces in DRC vary in terms of their geographical and epidemiological characteristics, their exposure to conflict, and support from development partners. Given that the management of the health system is decentralized, and that we have a relatively small sample, we do not attempt to explain the regional differences.

The study is not without limitations. First, Hawthorne effects may have improved provider behavior, making us overestimate the true quality of care. Second, our analytical benchmark was based on an ex-post coding following IMCI guidelines rather than expert opinion of other providers re-assessing children. Third, we did not collect information on previous diagnoses or treatments by other health centers or community health workers, and thus do not have complete information on patient conditions at the time of admission.

Fourth, the reliance on observed symptoms means that we cannot directly classify the disease severity of a relatively large number of cases. Given this limitation, the prevalence of severe disease in our study sample should not be interpreted as an estimate of the prevalence of severe disease among patients visiting health facilities in the DRC. While it is possible to compute the number of “potential severe diseases cases” based on the assumption all unobserved symptoms would be positive or negative, the true prevalence of severe disease is hard to establish in our data. Our approach of analyzing only classifiable cases could potentially bias our quality estimates upwards: by focusing only on the cases in which providers performed sufficient diagnostic testing to identify disease severity, we may be focusing only on the better providers. Given that many providers do not complete all the tests necessary to classify severe illnesses, patients in the missing data are more likely to be misdiagnosed and mistreated.

One of the key challenges with the results presented is that it remains largely unclear how improved compliance with IMCI guidelines can be achieved. Improving the supply of essential medicines is one necessary step: the national Service Availability and Readiness Assessment (SARA) in the DRC in 2014 found that only 50% of health facilities had amoxicillin in storage and only 23% had zinc [18]. However, the observed low quality of care is unlikely to be entirely explained by insufficient infrastructure or resources such as medical supplies or drugs. Indeed, in our study, being seen at a hospital rather than a health center far from guaranteed that severely ill children would receive needed in-patient care. And supply availability does not always translate into quality: a recent multi-country study found no significant differences in quality of care for sick children between more- and less-equipped health facilities [19].

Other studies have pointed to several potential alternative explanations for low quality. First, provider knowledge of evidence-based protocols may be an important limiting factor [3]. Of note, only 36% of providers taking care of patients in our severe disease sample reported being trained on the IMCI guidelines. Second, even when providers know the protocols, they may not implement them, exhibiting a “know-do gap” [2, 3, 20, 21]. This gap may be explained by several potential mechanisms including low provider effort, the high cognitive load health care providers experience, or providers’ disagreement with the clinical guidelines [20–22]. Health worker compensation in the DRC is highly fragmented: many health workers in public facilities are not on the public payroll, and a single health worker may receive payments from multiple funding streams without a consistent salary or strong link between payment and performance [23, 24]. This fragmentation may contribute to low levels of motivation to provide quality care. With an average of 4–5 under-5 patients per day, time constraints are unlikely to explain the low levels of compliance observed.

The solution is unlikely to lie with further scaling up implementation of current health worker training approaches. We found that IMCI training was not associated with improved quality of care for severely ill patients. Though early studies of IMCI training showed that it improved quality of care [25–31], the effects of this training may wane over time or may be minimal when the training is not the subject of a research study. Our results are in line with the findings of other studies showing that, generally, in-service training and supervision efforts have been insufficient to significantly improve quality of care in LMICs [32, 33]. This could point to a need to strengthen the quality of pre-service training systems.

Performance-based financing (PBF) programs, such as the one in the DRC, can be an effective way to improve quality of care. These programs provide reward payments to facilities and providers conditional on the quantity and quality of selected services. An evaluation of the PBF program in DRC is ongoing. Results from earlier pilots in other countries suggest they can have a meaningful impact on content of care [34–36].

Improved compliance with IMCI protocols may also require more focused quality-improvement programs, moving beyond training towards closer monitoring of performance along with feedback to providers. Community-based reviews of child mortality cases may be a promising way forward, bringing attention to the specific gaps that need to be addressed at a local level [37]. Several studies have also highlighted the potential for intensive mentorship or coaching programs to change behavior and improve quality [38].

## Conclusions

We found very low quality of care for children with severe febrile disease (including malaria), severe pneumonia, and severe dehydration in the DRC. The current RBF program in the study areas tries to directly address this by financially rewarding facilities where provider knowledge of IMCI protocols is high; it will be interesting to see whether such financial incentives are sufficient to increase protocol compliance in this setting.

## Supplementary information

**Additional file 1: Figure S1 and Table S1.** show results for patients with severe dehydration. **Tables S2 and S3.** show results for patients with severe febrile disease who were not tested for malaria as well as for those who tested negative for malaria.

## Data Availability

The datasets supporting the conclusions of this article are available by request from the World Bank microdata catalog, http://microdata.worldbank.org/index.php/catalog/2825.

## Abbreviations

DRC: Democratic Republic of the Congo
iCCM: integrated Community Case Management
IMCI: Integrated Management of Childhood Illness
ODK: Open Data Kit
ORS: Oral Rehydration Salts
PBF: Performance-Based Financing
SARA: Service Availability and Readiness Assessment
WHO: World Health Organization

## Footnotes

1 Since the data analyzed in this study were collected, there has been a reform in the administrative division of the DRC into provinces. We used the pre-2015 division of provinces in this analysis.
