# Supplementary material for: Quality of care for children with severe disease in the Democratic Republic of the Congo
Source: BMC Public Health. 2019 Dec 2;19:1608. doi: 10.1186/s12889-019-7853-3 (PMC6889659; doi:10.1186/s12889-019-7853-3)
Supplement: Supplementary file 1 — Additional file 1: Figure S1 and Table S1. show results for patients with severe dehydration. Tables S2 and S3. show results for patients with severe febrile disease who were not tested for malaria as well as for those who tested negative for malaria. [file 12889_2019_7853_MOESM1_ESM.docx]

**Quality of care for children with severe disease in Congo, DRC**

**Additional file 1**

Table S1: Diagnosis and treatment of severe dehydration

| Proportion of severe dehydration cases in which providers… | | | | | | | |  |
| --- | --- | --- | --- | --- | --- | --- | --- | --- |
|  |  | **Diagnosed with moderate or severe dehydration** | **Diagnosed with severe dehydration** | **Prescribed fluids** | **Prescribed antibiotics** | **Prescribed both fluids and antibiotics** | **Recommended**  **in-patient care (in the facility where the visit took place)** |  |
|  | *N* | *N (%)* | *N (%)* | *N (%)* | *N (%)* | *N (%)* | *N (%)* |  |
| All | 19 | 14 (74) | 7 (37) | 9 (53) | 10 (59) | 5 (29) | 3 (16) |  |
|  |  |  |  |  |  |  |  |  |
| Province |  |  |  |  |  |  |  |  |
| Bandundu | 5 | 3 (60) | 2 (40) | 3 (60) | 2 (40) | 1 (20) | 2 (40) |  |
| Equateur | 5 | 4 (80) | 2 (40) | 0 (0) | 3 (75) | 0 (0) | 0 (0) |  |
| Katanga | 2 | 1 (50) | 1 (50) | 1 (50) | 1 (50) | 1 (50) | 0 (0) |  |
| North Kivu | 2 | 1 (50) | 1 (50) | 2 (100) | 1 (50) | 1 (50) | 0 (0) |  |
| South Kivu | 5 | 5 (100) | 1 (20) | 3 (75) | 3 (75) | 2 (50) | 1 (20) |  |
| *p-value* |  | *0.546* | *0.943* | *0.847* | *0.132* | *0.558* | *0.479* |  |
|  |  |  |  |  |  |  |  |  |
| Urban or rural |  |  |  |  |  |  |  |  |
| Urban | 0 | - | - | - | - | - | - |  |
| Rural | 19 | 14 (74) | 7 (37) | 9 (53) | 10 (59) | 5 (29) | 3 (16) |  |
|  |  |  |  |  |  |  |  |  |
| Facility type |  |  |  |  |  |  |  |  |
| Hospital | 11 | 9 (82) | 4 (36) | 4 (40) | 6 (60) | 2 (20) | 3 (27) |  |
| Referral health center | 2 | 1 (50) | 1 (50) | 2 (100) | 1 (50) | 1 (50) | 0 (0) |  |
| Other health center | 6 | 4 (67) | 2 (33) | 3 (60) | 3 (60) | 2 (40) | 0 (0) |  |
| *p-value* |  | 0.619 | 0.926 | 0.97 | 0.321 | 0.625 | 0.309 |  |
|  |  |  |  |  |  |  |  |  |
| Training on IMCI protocols |  |  |  |  |  |  |  |  |
| Trained | 5 | 3 (60) | 2 (40) | 3 (60) | 3 (60) | 2 (40) | 1 (20) |  |
| Not trained | 14 | 11 (79) | 5 (36) | 6 (50) | 7 (58) | 3 (25) | 2 (14) |  |
| *p-value* |  | 0.447 | 0.874 | 0.953 | 0.728 | 0.566 | 0.779 |  |
|  |  |  |  |  |  |  |  |  |
| Provider type |  |  |  |  |  |  |  |  |
| Doctor | 10 | 9 (90) | 5 (50) | 5 (56) | 5 (56) | 2 (22) | 2 (20) |  |
| Nurse | 9 | 5 (56) | 2 (22) | 4 (50) | 5 (62) | 3 (38) | 1 (11) |  |
| *p-value* |  | 0.098 | 0.233 | 0.788 | 0.832 | 0.521 | 0.62 |  |
|  |  |  |  |  |  |  |  |  |
| Diagnosis |  |  |  |  |  |  |  |  |
| Dehydration, not severe | 7 | - | - | 4 (57) | 6 (86) | 3 (43) | 0.00 (0.00) |  |
| Dehydration, severe | 7 | - | - | 2 (33) | 3 (50) | 1 (17) | 0.14 (0.38) |  |
| Not diagnosed with dehydration | 5 | - | - | 3 (75) | 1 (25) | 1 (25) | 0.40 (0.55) |  |
| *p-value* |  | - | - | 0.612 | 0.259 | 0.426 | 0.898 |  |
|  |  |  |  |  |  |  |  |  |
| Number of severe disease classifications |  |  |  |  |  |  |  |  |
| 1 | 3 | 1.00 (0.00) | 0.33 (0.58) | 2 (40) | 3 (60) | 2 (40) | 0.00 (0.00) |  |
| 2 | 11 | 0.82 (0.40) | 0.36 (0.50) | 4 (44) | 6 (67) | 2 (22) | 0.27 (0.47) |  |
| 3 | 5 | 0.40 (0.55) | 0.40 (0.55) | 3 (100) | 1 (33) | 1 (33) | 0.00 (0.00) |  |
| *p-value* |  | *0.123* | *0.984* | *0.321* | *0.483* | *0.333* | *0.309* |  |

Notes: This table shows quality of care for the subset of patients who met the IMCI classification for severe dehydration based on observed symptoms. The columns represent different steps in providing quality of care for severe dehydration, and the rows represent different subsets of the data. The results shown are the number of patients and percent of patients in each subgroup for whom each step was taken (based on direct observation data and caregiver exit interviews). P-values were calculated using a chi-square test comparing means in the subgroups presented. There are two cases with missing information on the treatments given; we assume that this information is missing completely at random. Results for referrals to other health facilities are not shown because no patients with signs of severe dehydration were referred to another health facility.

Table S2: Diagnosis and treatment of severe febrile disease without malaria test

|  |  | Proportion of severe febrile disease without malaria test in which providers… | | | | | | |  |
| --- | --- | --- | --- | --- | --- | --- | --- | --- | --- |
|  |  | **Diagnosed with malaria** | **Prescribed antibiotics** | **Prescribed anti-malarials** | **Prescribed paracetamol** | **Prescribed the correct treatments (based on temperature)** | **Recommended in-patient care (where the visit took place)** | **Referred to another facility** | |
|  | *N* | *N (%)* | *N (%)* | *N (%)* | *N (%)* | *N (%)* | *N (%)* | *N (%)* | |
| All | 131 | 95 (73) | 51 (49) | 62 (60) | 34 (33) | 23 (22) | 8 (6) | 5 (4) | |
|  |  |  |  |  |  |  |  |  | |
| Province |  |  |  |  |  |  |  |  | |
| Bandundu | 37 | 28 (76) | 15 (47) | 22 (69) | 11 (34) | 8 (25) | 4 (11) | 1 (3) | |
| Equateur | 52 | 42 (81) | 17 (55) | 23 (74) | 9 (29) | 7 (23) | 2 (4) | 1 (2) | |
| Katanga | 14 | 12 (86) | 2 (14) | 6 (43) | 2 (14) | 1 (7) | 1 (7) | 0 (0) | |
| North Kivu | 6 | 2 (33) | 2 (33) | 1 (17) | 2 (33) | 0 (0) | 0 (0) | 0 (0) | |
| South Kivu | 22 | 11 (50) | 15 (71) | 10 (48) | 10 (48) | 7 (33) | 1 (5) | 3 (14) | |
| *p-value* |  | *0.008* | *0.015* | *0.021* | *0.099* | *0.235* | *0.672* | *0.126* | |
|  |  |  |  |  |  |  |  |  | |
| Urban vs. rural |  |  |  |  |  |  |  |  | |
| Urban | 12 | 9 (75) | 1 (12) | 3 (38) | 2 (25) | 1 (12) | 1 (8) | 0 (0) | |
| Rural | 119 | 86 (72) | 50 (52) | 59 (61) | 32 (33) | 22 (23) | 7 (6) | 5 (4) | |
| *p-value* |  | *0.841* | *0.032* | *0.188* | *0.445* | *0.425* | *0.738* | *0.473* | |
|  |  |  |  |  |  |  |  |  | |
| Facility type |  |  |  |  |  |  |  |  | |
| Hospital | 39 | 33 (77) | 14 (41) | 20 (59) | 10 (29) | 7 (20) | 5 (13) | 0 (0) | |
| Referral health center | 13 | 62 (70) | 6 (67) | 6 (67) | 2 (22) | 16 (23) | 2 (15) | 0 (0) | |
| Other health center | 79 | 59 (75) | 31 (51) | 36 (59) | 22 (36) | 14 (23) | 1 (1) | 5 (6) | |
| *p-value* |  | *0.288* | *0.368* | *0.906* | *0.642* | *0.967* | *0.016* | *0.184* | |
|  |  |  |  |  |  |  |  |  | |
| Training on IMCI protocols |  |  |  |  |  |  |  |  | |
| Trained | 39 | 27 (69) | 12 (43) | 15 (54) | 12 (43) | 5 (18) | 3 (8) | 2 (5) | |
| Not trained | 92 | 68 (74) | 39 (51) | 47 (62) | 22 (29) | 18 (24) | 5 (5) | 3 (3) | |
| *p-value* |  | *0.586* | *0.449* | *0.451* | *0.417* | *0.57* | *0.625* | *0.613* | |
|  |  |  |  |  |  |  |  |  | |
| Provider type |  |  |  |  |  |  |  |  | |
| Doctor | 31 | 20 (65) | 8 (32) | 17 (68) | 6 (24) | 5 (20) | 4 (13) | 0 (0) | |
| Nurse | 95 | 71 (75) | 39 (53) | 42 (57) | 25 (34) | 16 (22) | 4 (4) | 5 (5) | |
| Other | 5 | 4 (80) | 4 (80) | 3 (60) | 3 (60) | 2 (40) | 0 (0) | 0 (0) | |
| *p-value* |  | *0.51* | *0.074* | *0.619* | *0.158* | *0.589* | *0.184* | *0.379* | |
|  |  |  |  |  |  |  |  |  | |
| Number of IMCI-determined SDs |  |  |  |  |  |  |  |  | |
| 1 | 63 | 49 (78) | 15 (31) | 30 (62) | 14 (29) | 6 (12) | 4 (6) | 4 (6) | |
| 2+ | 68 | 46 (68) | 36 (64) | 32 (57) | 20 (36) | 17 (30) | 4 (6) | 1 (1) | |
| *p-value* |  | *0.197* | *0.001* | *0.583* | *0.352* | *0.029* | *0.912* | *0.148* | |
|  |  |  |  |  |  |  |  |  | |

Notes: This table shows quality of care for the subset of patients who met the IMCI classification for severe febrile disease based on observed symptoms and were not tested for malaria. The columns represent different steps in providing quality of care for severe febrile disease, and the rows represent different subsets of the data. The results shown are the number of patients and percent of patients in each subgroup for whom each step was taken (based on direct observation data and caregiver exit interviews). P-values were calculated using a chi-square test comparing means in the subgroups presented. There are 27 cases with missing information on the treatments given; we assume that this information is missing completely at random.

Table S3: Treatment of other (non-malarial) severe febrile disease

|  |  | Proportion of severe febrile disease with confirmed malaria cases in which providers… | | | | | |
| --- | --- | --- | --- | --- | --- | --- | --- |
|  |  | **Diagnosed with malaria** | **Prescribed antibiotics** | **Prescribed anti-malarials** | **Prescribed paracetamol** | **Prescribed the correct treatments (based on temperature)** | **Recommended in-patient care** |
|  | *N* | *N (%)* | *N (%)* | *N (%)* | *N (%)* | *N (%)* | *N (%)* |
| All | 27 | 14 (52) | 16 (62) | 12 (46) | 19 (73) | 4 (15) | 2 (7) |
|  |  |  |  |  |  |  |  |
| Province |  |  |  |  |  |  |  |
| Bandandu | 4 | 4 (100) | 2 (50) | 2 (50) | 3 (75) | 0 (0) | 1 (25) |
| Equateur | 6 | 2 (33) | 3 (60) | 2 (40) | 3 (60) | 1 (20) | 0 (0) |
| Katanga | 4 | 3 (75) | 3 (75) | 2 (50) | 3 (75) | 0 (0) | 0 (0) |
| North Kivu | 2 | 1 (50) | 2 (100) | 1 (50) | 2 (100) | 1 (50) | 1 (50) |
| South Kivu | 11 | 4 (36) | 6 (55) | 5 (45) | 8 (73) | 2 (18) | 0 (0) |
| *p-value* |  | *0.18* | *0.778* | *0.998* | *0.753* | *0.639* | *0.061* |
|  |  |  |  |  |  |  |  |
| Facility type |  |  |  |  |  |  |  |
| Hospital | 4 | 4 (100) | 3 (75) | 1 (25) | 2 (50) | 1 (25) | 0 (0) |
| Referral health center | 5 | 3 (60) | 5 (100) | 3 (60) | 5 (100) | 1 (20) | 2 (40) |
| Other health center | 18 | 7 (39) | 8 (47) | 8 (47) | 12 (71) | 2 (12) | 0 (0) |
| *p-value* |  | *0.083* | *0.089* | *0.605* | *0.241* | *0.613* | *0.005* |
|  |  |  |  |  |  |  |  |
| Training on IMCI protocols |  |  |  |  |  |  |  |
| Trained | 11 | 7 (64) | 7 (64) | 4 (36) | 7 (64) | 1 (9) | 1 (9) |
| Not trained | 16 | 7 (44) | 9 (60) | 8 (53) | 12 (80) | 3 (20) | 1 (6) |
| *p-value* |  | *0.328* | *0.858* | *0.412* | *0.543* | *0.481* | *0.792* |
|  |  |  |  |  |  |  |  |
| Provider type |  |  |  |  |  |  |  |
| Doctor | 3 | 3 (100) | 2 (67) | 2 (67) | 1 (33) | 1 (33) | 0 (0) |
| Nurse or other | 24 | 11 (46) | 14 (61) | 10 (43) | 18 (78) | 3 (13) | 2 (8) |
| *p-value* |  | *0.082* | *0.854* | *0.469* | *0.147* | *0.43* | *0.62* |
|  |  |  |  |  |  |  |  |
| Number of IMCI-determined SDs |  |  |  |  |  |  |  |
| 1 | 10 | 7 (70) | 4 (40) | 5 (50) | 6 (60) | 1 (10) | 1 (10) |
| 2+ | 17 | 7 (41) | 12 (75) | 7 (44) | 13 (81) | 3 (19) | 1 (6) |
| *p-value* |  | *0.159* | *0.08* | *0.767* | *0.385* | *0.591* | *0.707* |
|  |  |  |  |  |  |  |  |

Notes: This table shows quality of care for the subset of patients who met the IMCI classification for severe febrile disease based on observed symptoms, and tested negative for malaria. The columns represent different steps in providing quality of care for severe febrile disease, and the rows represent different subsets of the data. The results shown are the number of patients and percent of patients in each subgroup for whom each step was taken (based on direct observation data and caregiver exit interviews). P-values were calculated using a chi-square test comparing means in the subgroups presented. There is one case with missing information on the treatments given; we assume that this information is missing completely at random. The results for referrals are not shown because no patients in this group were referred to another facility.

Figure S1: Diagnosis of patients with IMCI signs of severe dehydration (N = 19)

Notes: This figure shows the diagnoses that providers gave to patients showing IMCI signs of severe dehydration, as captured through direct observation. The patients represented by the blue portion of the pie were diagnosed with severe dehydration. The patients represented by the green portion of the pie were diagnosed with a moderate dehydration. The patients represented by the pink portion of the pie were not diagnosed with dehydration.
